# IoT/Sensor-Based Infrastructures Promoting a Sense of Home, Independent Living, Comfort and Wellness

**DOI:** 10.3390/s19030485

**Published:** 2019-01-24

**Authors:** Joan Cahill, Raul Portales, Sean McLoughin, Nithia Nagan, Braden Henrichs, Sean Wetherall

**Affiliations:** 1 School of Psychology, Trinity College Dublin, Dublin 2, Ireland; 2 Oneview Healthcare, Blackrock Business Park, Blackrock, Co. Dublin, Ireland; rportales@oneviewhealthcare.com (R.P.); smcloughlin@oneviewhealthcare.com (S.M.); nnagan@oneviewhealthcare.com (N.N.); bhenrichs@oneviewhealthcare.com (B.H.); swetherall@oneviewhealthcare.com (S.W.)

**Keywords:** ambient-assisted living, ageing, residential care, wellness, care delivery, sensors, tablets

## Abstract

This paper presents the results of three interrelated studies concerning the specification and implementation of ambient assisted living (AAL)/Internet of Things (IoT)/sensor-based infrastructures, to support resident wellness and person-centered care delivery, in a residential care context. Overall, the paper reports on the emerging wellness management concept and IoT solution. The three studies adopt a stakeholder evaluation approach to requirements elicitation and solution design. Human factors research combines several qualitative human–machine interaction (HMI) design frameworks/methods, including realist ethnography, process mapping, persona-based design, and participatory design. Software development activities are underpinned by SCRUM/AGILE frameworks. Three structuring principles underpin the resident’s lived experience and the proposed ‘sensing’ framework. This includes (1) resident wellness, (2) the resident’s environment (i.e., room and broader social spaces which constitute ‘home’ for the resident), and (3) care delivery. The promotion of resident wellness, autonomy, quality of life and social participation depends on adequate monitoring and evaluation of information pertaining to (1), (2) and (3). Furthermore, the application of ambient assisted living technology in a residential setting depends on a clear definition of related care delivery processes and allied social and interpersonal communications. It is argued that independence (and quality of life for older adults) is linked to technology that enables interdependence, and specifically technology that supports social communication between key roles including residents, caregivers, and family members.

## 1. Introduction

### 1.1. Introduction to Study Problem

Ambient intelligence is a new paradigm in information technology aimed at empowering people’s capabilities by the means of digital environments that are sensitive, adaptive, and responsive to human needs and the presence of people [1,2,3]. Typically, context-aware technology is integrated in a person’s living environment. 

Assisted living/residential care settings are defined as group living environments for adults with disabilities and/or older adults who require assistance with at least one activity of daily living. In many cases, older adults may have some level of cognitive and functional decline. Assisted living facilities follow a social model of care (i.e, beyond bio-medical) which is predicated on the concept of ‘home’ and ‘resident engagement’. Importantly, it is expected that residents live in an environment that resembles home and enables personal autonomy and social connection.

Ambient assisted living (AAL) can be defined as “the use of information and communication technologies (ICT) in a person’s daily living and working environment to enable them to stay active longer, remain socially connected and live independently into old age” [4]. 

New technologies are being advanced to support the needs of older people living independently (i.e., at home) and in assisted living/residential care environments [5]. Generally, this involves the advancement of AAL/Internet of Things (IoT)/sensor-based infrastructures which make use of machine learning technology. These infrastructures connect a range of devices (i.e., TV, tablet, mobile phones etc.), sensors (environmental sensors and biosensors) and actuators as part of an IoT hub. The above technologies link to concepts of ambient assistive living and ‘any-every connectivity’ [6].

The purpose and functionalities of gerontechnologies are often led by the requirements of their social and caregiving environments [7]. Often, there is a mismatch between the proposed functionalities and end user intrinsic motivations and expected benefits [7,8]. As highlighted by Chen and Chan [8], this has an impact on user acceptance. If technologies are to be embedded in a person’s life, then they must be acceptable to that person [9].

Overall, the application of AAL technology needs to have meaning and value in the context of (1) the lived experience and diverse needs of older adults in residential care, and (2) the care processes and activities that underpin the management of resident wellness in this setting. To this end, the available technology requires interrogation in relation to (1) stakeholders needs, benefits, requirements and issues pertaining to ethics and acceptability. Furthermore, this technology is quite new, and basic research is required in relation to (2) investigating how AAL/IoT/sensor-based infrastructures might be designed to deliver on stakeholder needs.

The concepts and technologies arising from research pertaining to (1) and (2), require implementation at a residential care site (3). To be successful, the functionality must address organizational goals along with the needs of older adults and caregivers. In addition, the introduction of new AAL technology (i.e., quality improvement) involves change right across the care setting. Such technology transforms processes, role definition and how care will be provided. Implementation strategies (a) are typically multi-component and (b) must adapt to local contexts [10]. Such change takes time and is often piece-meal [11]. Potentially, the union of quality improvement science and human factors can produce safer and better solutions for healthcare [12].

### 1.2. Conceptualizing the Study Problem and Underpinning Theory

The starting point for conceptualizing the application of AAL is the experience of the older adult domicile in a residential care facility. According to research by the American Association of Retired Persons, nearly 90 percent of older adults want to ‘age in place’ [13]. The home is associated with many positives. Chief among them is the comfort of being in ones’ own environment and the associated implications of user control and privacy. However, research indicates that the home is not always the ideal environment for fostering independence and quality of life. Studies highlight the potential for social isolation [14] and disempowerment [15]. This often occurs in situations where the home is the site for medical treatment [16]. 

In the US, there is a strong trend in relation to older adults transitioning to retirement/assisted living communities [17]. In Europe, older people tend to live at home and move to residential care facilities at a more acute stage. Residential homes have been associated negative experiences including regimented routines, lack of freedom, reduced social connection [18], boredom and encroachments on privacy [19]. However, recently, there has been a ‘culture change’ in residential care, with more attention to social relationships, resident preferences and promoting intergenerational contact.

Although rewarding, the job of being a nurse and/or a care assistant is very demanding. Workload is high. Staff-to-resident ratios are lower than those at acute care environments [20]. Caregiver burnout is frequently reported [21]. Critically, staff burnout can adversely impact on care quality and safety. For example, research indicates that caregiver burnout directly impacts patient mortality [22]. Moreover, burnout increases the risk of neglect and abuse, especially in older populations [23,24].

Many assistive living facilities depend on a mix of both contract and permanent staff. As a result, it can be difficult to ensure consistency in relation staff training and care culture. Process compliance and resident reporting is closely monitored. Often, there is a conflict between resident reporting and providing direct personal care to residents [25]. Challenges to professionalism are frequently reported [26,27,28]. In such environments, resident safety depends upon open communication, trust and effective interdisciplinary teamwork [29].

Several accounts of ageing have been proposed. Biomedical models focus on the avoidance of disease and disability [30]. According to Rowe and Kahn [31], successful aging is multidimensional encompassing the avoidance of disease and disability, the maintenance of high physical and cognitive function, and sustained engagement in social and productive activities Similarly, psychosocial models focus on life satisfaction, social participation, functioning, and psychological resources [32,33].

The concept of identity has three pillars: the person, the role and the group [34]. Personal identity refers to the sense of self which is built over time. This includes the aggregate of characteristics by which a person is recognized by himself/herself and others, what matters to the person and their values [35]. Crucially, autonomy is central to personal identity [35]. Others define identity as the social position that the self both possesses and internalizes [36]. It is argued that individuals use their identities during their interaction with others. That is, they play roles [36]. Furthermore, membership in a community influences identity [36]. The pillars of role and group are fundamental to the notion of the relational self [37] and the allied concept of relational identity [37]. Relational identity refers to the part of the person’s identity that is based on their relationship with another person (for example, husband or wife) [37]. 

Rights are the fundamental normative rules concerning what a person is entitled to. Charters of rights for older adults living in care homes have been advanced both generally [38] and in the memory care context [39]. These charters highlight certain principles that should be considered in relation to the introduction of new AAL technologies. For example, resident consent in relation to the use of specific technologies/sensors and associated information captured, and the protection of the personal sphere.

The concept of autonomy refers to exercising individual choice, freedom of will, and assuming responsible for oneself [40,41]. As human relationships are based on mutual dependence and partnership [42], it is argued that autonomy should be conceptualized in the context of the relationships within which individuals are embedded [43].

According to biopsychosocial theories of health and wellness, the cause, manifestation and outcome of wellness and disease are determined by a dynamic interaction between biological, psychological and social factors. None of these factors in isolation are sufficient to lead definitively to wellness or illness. Instead, it is the interrelationships between all three pillars that results in each outcome [44,45]. 

Patient experience refers to how the patient feels as they undergo an episode of care [46]. The concepts of ‘resident experience’ follows from the concepts of patient experience. Resident experience pertains to the ‘lived experience’ of residents in assistive living facilities. It concerns their overall quality of life and level of fulfillment [47]. 

Patient engagement is defined as a patient’s own engagement in their health, their care, and their treatment [48,49]. It is argued that engaged patients are better able to make informed decisions about their care options [50,51]. Similarly, resident engagement pertains to the level of interest/participation that an older adult domicile in a residential care facility takes in their daily care and activities [52].

Patient-centered approaches have replaced physician centered care approaches [42]. Some argue that the concept of patient-centered care might be replaced with the concept of person-focused care [53]. More recently, there has been a move towards relationship-centered care [54,55,56]. As human beings are social beings [57,58], fostering and maintaining positive social relationships is essential to well-being. Furthermore, this positively impacts on health outcomes.

### 1.3. Paper Overview

This paper presents the results of three interrelated studies concerning the specification and implementation of AAL/IoT/sensor-based infrastructures to support (a) resident wellness and (b) person-centered care delivery in a residential care context (i.e., community dwelling and not a private home). Overall, these three studies form the basis of an overall product development project, involving collaboration between researchers at Trinity College Dublin and at Oneview Healthcare.

The first study pertains to human factors research concerning the specification of new AAL technology, in relation to the user requirements of residents and caregivers. The second study reports on the advancement of an AAL product, as part of a commercial product development project. This study was undertaken in parallel to the first study. Accordingly, the outputs of Study 1 were iteratively fed into Study 2, on an ongoing basis. The third study focuses on the implementation of the emerging product concept, at two customer sites.

First the materials and methods are introduced. This includes an overview of research objectives, a summary of the three studies, and a detailed breakdown of the methods used in each of the studies. Following this, the results of the three studies are presented. The results are reported in relation to a series of themes which have emerged from an analysis of findings across the three studies. In relation to study 1a and b, the primary focus is on reporting wellness management concepts and processes. It should be noted that specific findings pertaining to stakeholder need (residents, care staff and families) and the user interface design of tablet solutions for residents and caregivers, has been described in more detail in other papers [59,60,61]. The proposed solution is then introduced. This includes a description of the overall sensing solution in terms of three proposed sensing dimensions, the resident record/profile, the process flow from a care-giver and resident’s perspective, and the wellness indicator. Following this, the technical dimensions of the proposed solution are reviewed. This includes a description of the technical architecture and data platform, data analytics processes, the implementation technology, aspects pertaining to security and scalability, and a description of the sensor technology for customer 1. The findings of the three studies are then discussed including study limitations and areas for further research. Finally, some conclusions are drawn.

## 2. Materials and Methods

### 2.1. Research Objectives

The overall objective of the three studies is to identify and validate the requirements for new technology enabling resident wellness and person-centered care delivery, in a residential care environment. Primarily this concerns the requirements of two key stakeholders; namely (1) older adults/residents and (2) care givers.

### 2.2. Overview of Studies

**Figure 1:**
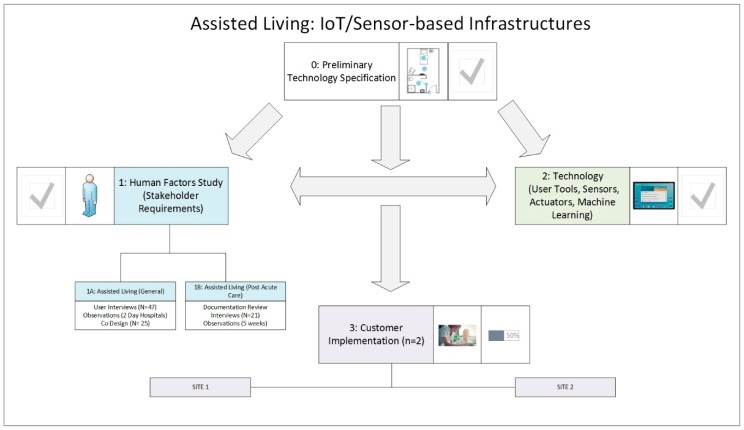
Overview of the three studies.

As indicated in Figure 1 below, three inter-related studies have been undertaken. Prior to the commencement of the human factors research (i.e., Study 1), a preliminary ‘assisted living’ (AL) product was advanced at Oneview Healthcare (see Figure 1, study 0). This involved the initial specification of the technical architecture and associated sensor kit and tablet solution [62]. As indicated in Figure 1, this preliminary research was further advanced in two parallel studies, Study 1 and Study 2, and then implemented at two customer sites (i.e., Study 3).

Study 1 comprises two interrelated human factors studies. Study 1a pertains to the requirements for new AL technology in different contexts (i.e., lifespan perspective). This includes private dwellings, assisted living environments and residential care environments. Study 1b pertains to the requirement for new AL technology in a post-acute care service. In relation to both studies, the human factors design approach is premised on the assumption that the solutions for older adults, caregivers, family members and other relevant actors are necessarily interrelated. To this end, a stakeholder evaluation approach [63] to requirements elicitation and user interface design was adopted. Both studies combine several qualitative human–machine interaction (HMI) design frameworks/methods. In relation to needs analysis, the methodological approach is underpinned by realist ethnography [64,65,66,67]. In relation to requirements specification, prototyping and design/evaluation activities, the methodology combines aspects of ‘personae-based design’ [68,69] and ‘participatory design’ [70].

Study 2 focused on the advancement of an AAL product for the residential care market. This includes the specification of the product concept, stakeholder requirements, the product’s technical architecture and associated software development. 

Study 3 is currently ongoing and involves action research pertaining to the implementation of the proposed product with two different customers (i.e., assisted living facilities) in Australia. The two customers have different objectives and requirements, different levels of technical maturity and different technical infrastructures (different integration requirements). In both cases, residents include a mix of low, mid and high dependency older adults, requiring a spectrum of assistance in terms of supporting the activities of daily living. A significant proportion of residents have some form of functional and cognitive decline (including Dementia). 

In relation to Customer 1, the initial implementation focuses on one demonstration site. This involves a newly built 120-bed facility, with an existing ambient and health sensor solution in place. This study is being led by the company’s chief information officer (CIO). The project team includes staff with clinical, technical and business transformation/change management expertise. The objective is to enhance the resident experience (integrating IoT with existing sensors provided by a different vendor), and to digitize the clinical and administrative workflows, at the facility. Tablet solutions are also being advanced for the resident. Currently, the technical solution has just been installed. A period of implementation, staff and resident training, review and evaluation is planned. Pending feedback, certain user interface design features/functions may be updated. Further, pending success in relation to phase 1 delivery, a second implementation phase will focus on retrofitting older sites with this technology.

In relation to Customer 2, the study is being led by an interdisciplinary team comprising the national systems manager, a project manager, a clinical lead and a national trainer. The project team includes staff comprising clinical, technical and business transformation/change management expertise. In relation to Customer 2, the initial implementation phase focuses on enhancing/replacing their existing clinical management system (CMS). This involves improving existing clinical workflows and enabling new points of access to these workflows (i.e., introducing new tablet and desktop-based solutions for nursing and administrative staff). Currently, customer requirements have been defined, and software development activities are underway. Software development will bridge those gaps identified between the existing Oneview assisted living product (i.e., clinical/caregiver focus), and the customer’s existing CMS. Once developed, the CMS will be implemented at a single site and any issues identified. The product will then be refined and further implemented at other sites. Pending success, it is anticipated that a second phase will focus on further enhancing the resident experience to include tablet solutions for residents and, potentially, ambient sensors.

### 2.3. Stakeholder Evaluation Approach and Innovation

Collectively, the three studies apply a stakeholder evaluation approach [63] to requirements elicitation and user interface design. This is the gold standard for human factors action research pertaining to new technology development. Overall, this stakeholder evaluation approach has spanned formal human factors research activities with a stakeholder panel (i.e., studies 1a 1b), ‘customer insights’ analysis following Request for Information (RFI)/Request for Proposal (RFP) processes with numerous aged care providers in the US and Australia (i.e., Study 2), and customer co-design workshops (i.e., Study 3).

In relation to studies 1a and 1b, interviews and observations have been undertaken with stakeholders to identify requirements. In parallel, the product team, have engaged in requirements elicitation activities as part of Study 2. Health and aged care industries typically follow a procurement via tender process. Product and commercial teams have worked together to track and respond to relevant RFI/RFP processes with numerous aged care providers in the US and Australia. Product have used the requirements listed in these tender processes as ‘customer insights’ about what the industry is looking for from their technology partners.

In relation to studies 1a, participatory co-design sessions have been undertaken with stakeholders to define the user interface design requirements for relevant caregiver and resident solutions. Study 2 has involved software development in relation to these prototypes. 

The product concept and associated requirement specifications and prototypes have been further refined as part of Study 3. As detailed below, the proposed software prototypes were reviewed in a series of workshops with customers, to define specific customer requirements and user interface workflows/design features. This is described in more detail below.

### 2.4. Study 1a

The purpose of this study was to identify and validate the requirements for new technology supporting wellness, independence and social participation for older people domiciled in residential homes and/or assisted living communities. In terms of scope, the primary focus was on the resident experience and the associated resident solution. Solutions for other stakeholders (i.e., nurse, care assistant and family) are being advanced for the purpose of (1) complementing workflows associated with the resident solution (for example, a nurse accessing a resident wellness survey), and (2) providing task support for these other stakeholders in relation to addressing resident wellbeing (for example, a nurse viewing resident profile information and/or reporting on resident wellbeing) [59,60,61]. 

In line with Wenger’s approach [71] the stakeholder evaluation approach involves the active and ongoing participation of stakeholders throughout the project. As part of this, a ‘community of practice’ [71] comprising internal and external stakeholders was advanced. 

In relation to needs analysis, 47 interviews were undertaken (external stakeholders: N = 38, internal stakeholders: N = 9). This includes interviews with older people living independently, aged care nurses, family members, volunteers and experts in ageing and dementia. Preliminary observations were undertaken in two public day hospitals providing care for older people in the community. Initial observations were undertaken at a residential home. Following the analysis of field research findings, a Version 1 prototype of the resident and nurse/caregiver solution was developed for use in the co-design activities. Five phases of co-design/evaluation were then undertaken (Phase 1, N = 6, Phase 2, N = 5, Phase 3, N = 5, Phase 4, N = 5 and Phase 5, N = 5). 

The first phase of co-design/evaluation focused on eliciting stakeholder feedback regarding the high-level product concept for the resident, caregiver/nurse, family and administrative assistant applications. In advance of viewing the prototypes, participants reviewed a short Microsoft power-point presentation which provided a background to the research and preliminary findings, a summary of the different applications and functions, and an example persona. The review/co-design of prototypes was then undertaken. The initial prototypes were demonstrated to stakeholders using a laptop computer. The second phase of co-design involved the same procedure as phase 1. However, the second phase of co-design focused on the resident and nurse applications only. The third phase of co-design involved the same procedure as phase 1. However, this phase focused on the nurse applications only. The forth phase focused on refining the concepts, processes/workflows and specific UI design for both the resident and nursing applications. The fifth phase focused on the nurse applications only. The primary focus was on (a) evaluating those functions which pertain to resident wellness and (b) exploring how best to manage issues around reduction in human contact/optimizing human contact in care delivery.

Older people, especially those living with cognitive impairment are a vulnerable group and their dignity, rights and privacy must be safeguarded. The study has been conducted in accordance with the principles of the Declaration of Helsinki (as amended in 2013) [72]. Research methods have received ethics approval from the School of Psychology, Trinity College Dublin, Ireland. Appendix A provides a summary of research phases and activities and what has been achieved. For more information on methods, please see Appendix B.

### 2.5. Study 1b

The purpose of this study was to identify the requirements for new technology to support patient experience, patient reablement, patient-centered care, and staff professionalism at a post-acute care service/hospital. This was a preliminary and indicative study. It is anticipated that the findings of this study will be used to identify a case for future technology transformation at the service. Study 1b has involved the participation of both patients and staff at the service. Research has involved documentation analysis, observations of staff (10 half days, elapsing over 5 weeks), interviews with nursing/care staff (N = 20), and interviews with patients (N = 11). Following an analysis of field research findings, early stage prototypes of several new technology concepts for different stakeholders have been advanced. This includes preliminary concepts for an interactive whiteboard at the nurse’s station, a wall mounted display outside the patient’s room, a caregiver app (accessible using a tablet device), a nursing app (accessible using a tablet device) and a patient app (accessible using a mobile phone). This study received ethics approval from the Institutional Review Board of a Dublin-based hospital, along with the ethics committee of the School of Psychology, Trinity College Dublin, Ireland. 

### 2.6. Study 2

Study 2 has involved applied research undertaken by the ‘senior living’ product development team at Oneview Healthcare. Overall, the product development approach has followed SCRUM frameworks. SCRUM is a lightweight, iterative and incremental framework for product development [73,74]. In keeping with AGILE approaches, it promotes sustainable development and satisfying the customer through early and continuous delivery of software [75]. 

Product development has involved two high level strands of research, namely (1) product envisionment (i.e., product vision and rationale,) led by the product owner, and (2) software development undertaken by a team of software developers, following SCRUM approaches. 

Strand 1 (production envisionment) has been closely integrated with Study 1 (i.e., human factors research). Accordingly, the outputs of Study 1 (i.e., user requirement specifications, wellness management concepts and user interface design prototypes for different stakeholder tools) have been considered in terms of the advancement of the product concept. 

Overall, product development activity has involved the following tasks:

1. Literature review/market scan in relation to customer needs, technology trends and available technology.
2. Evaluation of the current state of the art in relation to sensor kits and IoT architectures.
3. Evaluation of prospective customer requirements, including technical and integration requirements (i.e., tender documentation).
4. Specification of the preliminary product vision, benefits and end user requirements.
5. Analysis of wellness management concepts, resident and caregiver user requirements (outputs of Study 1).
6. Definition of stakeholder requirements (outputs of study 1).
7. Specification of technical architecture.
8. Translating of requirements into user stories (outputs of study 1).
9. Prototyping and design, including an analysis of user interface design prototypes for residents and caregivers as emerged in human factors research (outputs of study 1).
10. Software development.
11. Software testing.
12. Product demonstration and evaluation.

The initial product proposal followed an analysis and synthesis of diverse information sources including information pertaining to competitor products, tender documents, Gartner reports, information from research partners and technology trends information. 

Product development had taken aspects of design thinking [76], lean startup [77] and rapid prototyping methodologies [78] to get the message of the product ethos and architecture to potential customers as early as possible. These product development methodologies focus on obtaining quick feedback from customers and iterating in a fast and low-cost way. Critically, iterations occur on code-free design prototypes, before developers take them into a development cycle.

In line with an AGILE product methodology, high level features are captured as an ‘Epic’. The purpose of the epic is to understand the high-level user value that a feature will deliver. The epic is refined to establish the ‘why’ (i.e., why it should be built). The ‘why’ is articulated in terms of customer, business and user value. Next, a technical architecture review is undertaken, to establish the ‘how’ (i.e., how it should be built from a technical perspective). At this stage, the epic is ready to be distilled down in the ‘user stories’ [79]. The product owner performs this ceremony with the product development team to ensure that the story is ‘ready for development’. Each user story follows the ‘INVEST’ method, so that it is: independent, negotiable, valuable, estimable, small and testable [79].

### 2.7. Study 3

In general, the action research methodology has involved several steps. The product team first reviewed a high level functional specification of the existing technology at the residential home, as provided by the customer.

The existing wellness management product (i.e., comprising different tablet solutions for residents and care-givers, a sensor-kit, data analytics, and solutions for administrative staff) was then reviewed by key members of the relevant project teams (Customer 1, N = 5, Customer 2, N = 6). As part of this, the customer provided preliminary feedback about end user need, identifying where the product addressed key requirements, and specific gaps. 

Following this, workshops were undertaken with clinical and administrative staff, to process map the existing care and administrative processes (Customer 1, N = 5, Customer 2, N = 6). In both cases, the workshops reflected a continuous discovery framework and elapsed over several weeks. As such, an iterative feedback loop was established between the customer and the product development team. 

A detailed review and evaluation of existing technology infrastructure and integration requirements was then undertaken with the customer’s IT team. The output of the above activities was analyzed by the product owner. The objective was to translate existing workflows into epics and user stories. 

A preliminary statement of work was then documented by the product owner detailing the scope, expectation and requirements on both parties in relation to the successful delivery of the product. This was signed off by the customer.

Following SCRUM methods, user stories were then documented. Software development was then undertaken in relation to the user stories. 

In relation to customer 1, the initial technology has just been installed at one site. In relation to customer 2, software development is still underway, and customer implementation is pending.

## 3. Results

### 3.1. Overview of Findings

This section presents an integration of findings across the 3 studies. As indicated in Table 1 below, the findings are grouped into a series of themes and sub-themes. As demonstrated in Table 1, there is considerable overlap in relation to focus of study 1 and study 3, and the focus of study 2 and study 3. 

The specific themes and sub-themes follow from the research focus of the three studies. In relation to the themes pertaining to stakeholder need/human factors requirements (i.e., 3.2 to 3.6), design (3.9) and managing change (3.10), the themes are derived from the data frames used in the two human factors studies (i.e., parent and child nodes corresponding to research questions defined for studies 1a and 1b). In relation to 3.7, the subthemes were selected so that general findings and findings pertaining to the three dimensions of the sensing framework (i.e., resident, environment and care delivery) could be easily distinguished.

**Figure 2:**
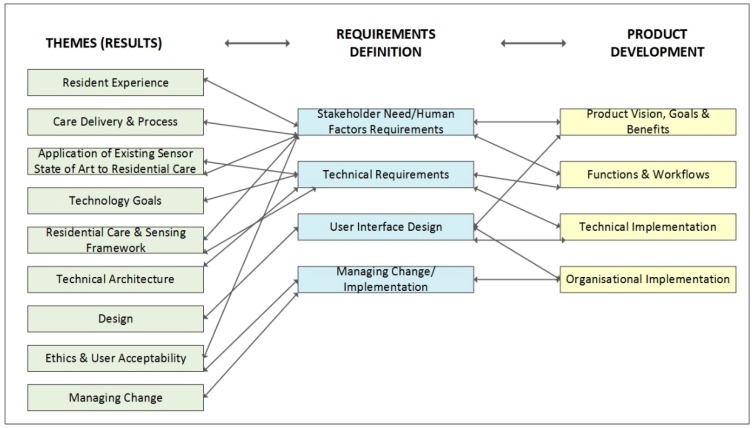
Themes, requirements definition and product.

As indicated in Figure 2 below, each of the themes is also associated with a classification in terms of requirements. Four types of requirements are specified. This includes stakeholder need/human factors, technical, user interface design and change management. This is turn is linked to different areas of product development, namely, production vision, goals and benefits, functions and workflow, technical implementation and organizational implementation. As shown in Figure 2, there is much crossover in terms of themes and requirements definition, and requirement definition and product development.

### 3.2. Resident Experience

Home

All participants referred to the concept of ‘home’. In relation to Study 1a and 1b, all participants reported on a preference to remain at home. In relation to Study 1b, participants expressed a desire to return home with a care package, as opposed to transitioning to long-term care. It was noted that a home is more than a physical space. As stated, ‘it is a feeling’.

Residential Homes

Participants differentiated between the experience of living at home (i.e., private home) and living in a residential home (i.e., community dwelling). According to participants, residential home differs to a private home on several levels. This includes:

- Other residents are domiciled there.
- Much of the environment is shared with others (for example, corridors, communal/social spaces, eating areas and outdoor areas).
- Care is provided by formal caregivers who ‘work’ at the residential home.

All participants observed that a residential home should function like a home.

Comfort

Older adults, care-givers and family members referred to the concept of ‘comfort’ and ‘being comfortable’. This feeling was described on two levels: in relation to (1) the comfort provided by the physical environment, and (2) the perceived social comfort provided by the residential environment. 

In relation to the former, participants referred to having control over certain aspects of their room. This includes room temperature, lighting, the opening and closing of windows and doors, the layout of the room/space and the style of furniture. Much of the environment is shared with others (for example, corridors, communal/social spaces, eating areas and outdoor areas). This has implications in terms of privacy and control. Participants reported an expectation that concepts of privacy and control might vary according to the specific physical space: (1) the resident’s room (full control to meet own comfort requirements), and (2) communal/social spaces (no expectation of personal control). 

In relation to social comfort, this includes being at ease socially and emotionally, and having a sense of connection with others in the care community (i.e., other residents and staff). Participants reported an expectation of being able to engage in activities with other residents. Furthermore, they reported an expectation that caregivers might get to know the residents as people and develop interpersonal relationships. As reported by caregivers, what is important is that residents have ample opportunities to experience and pursue meaningful social relationships. For some, this may involve meaningful connections with only a small number of family members and staff. Evidently, a healthy social life tends to vary from person to person, and in different circumstances (i.e., illness, bereavement etc.). Participants indicated that older adults might benefit from coaching in relation to fostering and maintaining identity and social participation and that, potentially, the technology might support this.

Resident Experience and Engagement

Overall, the concepts of resident experience and engagement are properly defined in the context of certain key psychosocial dimensions of resident wellness, and specifically, in relation to the quality of social and care relationships. This includes multileveled feelings and experiences of having an identity which is recognized and valued by oneself and others, having a sense of purpose and a role, obtaining meaning and value in daily routines/activities, identifying and relating to other residents and care givers, being part of a community, having a voice in relation to care decisions, and having confidence and trust in care-givers (personal communication).

Resident States

Participants provided feedback about different resident states to be promoted, managed/mitigated and avoided. Key states to be promoted include resident autonomy, social participation, wellbeing and purposeful activity. These are reported in more detail in [48,49,50]. 

Identity and Social Participation

Participants suggested that the sensor and tablet kit be used to promote social participation. The technology might provide caregivers with relevant information about the resident’s identity and personal history, to support meaningful connection and conversation. The technology might also be used to promote social activity for residents along with opportunities for self-growth (for example, getting information about how to start new activity). Furthermore, the technology might enable residents to be more proactive in relation to managing their own identity and social relationships (i.e., enabling organically occurring social relationships). Moreover, the technology might notify caregivers if there are changes in resident social participation. For example, the sensors could track time spent in their room, attendance at events, level of social activity, and notify care-givers and family members if there is a potential for neglect (i.e., not doing normal activities or reduction in activities). 

### 3.3. Wellness Management and Care Delivery

Scope of Wellness Management

Overall, participant feedback indicates that care delivery is multi-dimensional, spanning the behavior and actions of care staff in relation to the enactment of certain core care processes. The key care processes include: (1) pre-admissions (sharing past assessment and profile information information), (2) admissions, (3) initial assessments, (4) care planning, (5) daily care delivery and reporting, (6) adverse events reporting and (7) discharge/end of care. It was also noted that care delivery also includes the underlying care approach/ethos (i.e., person-centered care, promoting social participation and enablement and promoting family participation), at the residential care facility. Care givers notes that in the current paper-based process, resident care information is often disconnected.

Wellness and Stability

It was observed that, primarily, care tends to focus on the physical pillar. However, more recently, there has been increased attention to psycho-social wellness. All participants attributed importance to the psycho-social pillar. It was noted that concepts of stability are used in relation to establishing a resident’s baseline, and assessing whether there are changes to the baseline. All participants noted the importance of providing direct personal care to the resident.

Participants noted that for each pillar there are lots of different factors that might be tracked. Furthermore, the inter-relationship between pillars and factors must also be considered. Participants highlighted certain specific factors in relation to the three pillars that should be monitored or reported on (i.e., either via sensors, or human mediated via reporting tools/tablets used by residents, family members or care-givers). This includes: resident location, resident stability/health, mood, sleep, level of activity, pain, level of human contact, status of care received and level of social participation. A full list is provided in Appendix C. Participants noted that it might not be necessary to go into minute detail on all factors. It was suggested that the relevant subset of factors to track for a given resident, might be defined at the admissions stage.

Participants noted the following points in relation to interpreting signals (changes in wellness pillars and associated factors)

- Change to any one factor/specific pillar will influence the others
- Change to any one factor/specific pillar might be enough to initiate response/call to action
- Individual differences should be considered
- If there a reason for change, then this should impact on the care response (i.e., monitor or take action)

Wellness Communications

Participants noted that the scope of wellness communications is quite broad and includes (1) verbal communication between residents and family members, between residents and care-givers, and between residents (i.e., social activity), (2) resident physical behavior and non-verbal communication (i.e., fidgeting, taking exercise, wandering, nodding head, knitting etc.), (3) specific social behavior (attending community events, talking with family and caregivers), (4) making changes to room settings (door open/closed, adjusting heating), and (5) specific care communications between residents, care-givers and family members regarding the state of the person and care delivery. For examples of these in relation to specific stakeholder profiles, please see Appendix D. In this sense, all behavior/activity and associated communications around this, can be interpreted as ‘a wellness signal’. Participants suggested that sensors might be used to gather information about the above signals.

### 3.4. Residential Care and Sensing Framework

Overall, participants indicated that sensing might encompass three dimensions. This includes:

1. State of resident (health and wellness).
2. State of environment (i.e., resident’s room and other areas and associated implications for state of person and/or activity).
3. State of care delivery (i.e., medications taken, Activities of Daily Living (ADL) support, level of care contact, if report due).

The proposed sensors measure a range of ‘signals’ that can be ‘sensed’. Information pertaining to these signals might be gathered, integrated and interpreted. Following this, relevant actions might be taken, either automatically (i.e., at level of room—for example, changes to room temperature), or following review by a person/caregiver (i.e., care provision, new care intervention etc.). Participants suggested that in relation to (1) and (3), information might be communicated to care-givers, who would be responsible for taking remedial actions in relation to resident wellness. In relation to (2), it is expected that this action would be automated (i.e., change room temperature etc.). Furthermore, information gleamed from sensors in relation to (1) and (2) might then be co-related, and changes made at the level of (2) and (3). The outcome of such actions might also be monitored to assess if the intervention is successful, or if further interventions and/or health assessments (and associated changes to care plans) are required.

### 3.5. High-Level Technology Goals

Participants suggested a range of high level goals for the technology. These are detailed in Table 2 below.

### 3.6. Ethics and User Acceptability

The application of passive technology (i.e., passive sensors in beds and activity monitoring sensors) to monitor resident safety, prevent falls and raise alarms was welcomed by all. Further participants noted that they wander management technology (i.e., door sensors and sensors in the environment), would be acceptable, if this meant that they could move around freely. Participants liked the idea that from a resident perspective, much of the technology might be happening in the background (i.e., bed sensors and sensors on windows/doors). All participants emphasized the importance of upholding resident rights and dignity. It was agreed that personal and medical information should be protected. Critically, this information should not be shared with others without permission. 

One of the key issues reported by all participants, is that the introduction of this technology may lead to (a) a reduction in human contact, and (b) inferior quality care. All participants highlighted the importance of in person care (i.e., person to person communication). As stated, it is important to maintain the human element of care. This is characterized by human presence, responsive communication and empathetic communication. It was noted that if monitoring is automated, and in person contact mostly initiated by electronic help requests, then this might become quite isolating for residents. For example, it might result in situations where residents only receive in-person contact if they request contact/help.

One of the benefits of such a system is that it might be used to build a picture of what is normal for a person (i.e., baseline context), so that any changes from this baseline might be easily flagged and interpreted by caregivers. For example, over time, the system (i.e., triangulation from sensors, nurse reports, resident surveys) might build a picture of a person’s typical sleep patterns (i.e., two bed exits during the night, and 6 h sleep). 

Care-givers also reported concerns about staff being monitored (i.e., room sensors). Also, it was observed that although the intention might be to enable more time for person centered care, over time, this technology might be used to reduce staffing numbers/costs.

### 3.7. Application of Existing Sensors ‘State of the Art’

General

Existing sensors may be simplistic in their software and data capabilities, requiring more analysis to be done by the platform. Newer sensors may be intelligent, with their own machine learning algorithms running ‘on the edge’, sending more robust information rather than just data to the platform. For example, a simple door sensor may send an event when the state changes between open and closed. More intelligent sensors such as smart shoe sensors or thermal cameras that identify people, position, and falls will have applications and potentially machine learning on the devices themselves. As such, they do not send the raw data from the sensors, but rather the output of running that data through the application.

Sensors and Monitoring Resident

Presence sensors can be combined with other sensors to provide greater awareness as to the resident’s state. For example, when combined with the bed and light sensors, it is possible to detect that the resident has woken up and is moving around the room. This information can be used to automatically trigger the lighting systems to switch on to prevent the resident from stumbling over furniture. Combined with the door sensor, it is possible to detect when someone enters or exits the room. This information can be used to monitor time outdoors. Furthermore, if the resident is at risk of wandering to an unsafe area, we can notify a caregiver and/or lock specific access points. Moreover, in combination with using machine learning, we can track and monitor the activity habits of the resident and notify the care team if the amount of activity has decreased (i.e., if the resident spends a lot more time in a sedentary position, for example, sitting and watching TV).

A pressure sensor can be used to measure activity, specifically when combined with presence and bed sensors.

Bed sensors can provide an accurate measure of sleep time and sleep interruptions, which is an essential indicator of resident wellbeing. Using machine learning we can easily detect anomalies on the sleep patterns (i.e., time in bed and interruptions etc.), to identify the causes/contributory factors to sleep disruption, and to make changes, so that these problems are addressed before the resident’s situation worsens (i.e., predictive risk management). Furthermore, over time and in combination with machine learning, data from the bed and presence sensor can be used to understand when a person usually goes to sleep, and how it correlates to the quality of the sleep. Using that information, we can suggest the start of a sundown routine that can include soothing music and dimmed lights (potentially using appropriate colors) to help the resident stay in synch with these cycles. Similarly, we can understand when it is best to start a ‘get up’ routine that triggers lighting at the right time during the sleep cycle.

Door and window sensors are mostly used to determine the state of the room, but they can also give insights regarding the resident state. When combined with presence, the door and window sensors can give us an idea of the level of activity within the room, and how the person uses the space (where tends to spend time—for example, sitting on couch in living space, or sitting in bed).

Global Positioning System (GPS) insoles are especially useful when the resident is at risk of wandering or running away, since it can be used to locate the resident.

Traditionally health monitoring is performed by the care team and recorded on paper. However, with more modern technology, a resident can proceed with a self-assessment and the data is directly sent to the system. This information can also be co-related with information other sensors, to inform resident wellbeing assessments.

Sensors and Monitoring of the Environment

Temperature sensors can be linked to a humidifier/dehumidifier to keep the humidity of the room in the optimal range for the resident. Furthermore, machine learning techniques can be used in combination with information from temperature and other sensors, to identify wellness patterns (for example, the influence of temperature/humidity on sleep patterns). This information can be used to identify optimum room settings (i.e., temperature, heating, humidity), which deliver the best outcome for the resident.

Window and door sensors can be linked to security systems (i.e., not have doors open at night). Window/door sensors can be linked to room heating systems, to switch off heating when the room is empty, or to boost heating when a window is open. When combined with the presence sensor, this can be used to obtain information about the activity habits of the resident and automatically alert the care team if the amount of activity or time indoors or outdoors decreases. 

Harmful Gas/C02 sensors can be linked to actuators to open the windows or activate fans to clean the air inside.

Light sensors can be used to provide a good amount of light through the day, using it to regulate the intensity of the light bulbs automatically (i.e., if it is a very dark day outside). This sensor can be linked to a presence sensor, so that this is only activated when the resident is in the room. On the actuator side, room lights can be turned off automatically when the resident leaves the room. Further light sensors can be co-related to mood (see next section). Moreover, lights can be configured to match the resident’s night-time/ sundown routine (i.e., slowly dimming over evening).

A microphone can be used to detect if the environment is noisy or if there is a sudden impact that can indicate a fall. It can also be used to infer mood based on the speech patterns (for example, detecting sound/screams if the resident is agitated or angry.). Some microphone systems also come with speakers (for example, Alexa or Google Home), and can be used to provide music, white noise or other relaxing sounds, if sub optimal mood states (i.e., agitation, anger) are detected.

Sensors and Monitoring of Care Delivery

Staff members can swipe a card (RFID) when entering a room. The system then knows when they enter and exit each room. This can be used to track how long a person has been visited for and when correlated with other sensors, it can be used to measure the impact of this visit on the resident’s wellbeing. In addition to the benefits for the resident, this also provide metrics for the care team that can help them optimize their time and provide better care.

### 3.8. Design and Wellness Indicator

All participants stated that the tablet solution would need to be very simple (i.e., not providing too many options and/or too much information) and should adapt to age related changes. It was also noted that many people in residential care have early cognitive decline and/or dementia. As such, their ability to interact with tablet systems might be limited. 

It was suggested that relevant tablet solutions might provide visual feedback as to the wellness state of the resident. This would be useful for both residents, caregivers and family members.

### 3.9. Technical Architecture

The technical architecture should support enterprise level scalability (i.e., there may be hundreds of thousands of active daily users in a single region). The platform should be able to abstract information from any sensor. Both customers highlighted the importance of a stable and reliable platform. The sensors should report to local IoT gateways and report back to a cloud-hosted platform. 

It was suggested that the sensors need to be Wi-fi enabled and easily connect to the facilities Wi-fi network, with very little bandwidth requirements. As stated, Wi-fi allows for easy retrofitting, as opposed to wired (which requires running cables which is both expensive and difficult when residents are in situ in rooms). If using Wi-fi, a reliable/robust backup solution is required (i.e., backup is required to ensure continuity in situations where the Wi-fi fails). 

Furthermore, it was recommended that all analytics and AI algorithms be built into the cloud application service. Although it is possible to do analytics on the edge (i.e., locally), and this approach is likely to be adopted in the future, this technology is currently not stable enough.

All resident data should be protected and conform to relevant jurisdiction’s data protection guidelines.

It was recommended to utilize the existing power connections for the traditional PIR (passive infrared sensor), hence minimizing installation time and requiring no additional power connections.

The platform for detecting movement needs to be very accurate. In relation to movement, it requires a base algorithm that caters for all movement patterns and a variety of positions, postures and sizes/types. Specifically, the sensor needs to be able to detect different positions—for example, standing, sitting and sleeping positions. Also, it needs to detect a hunched adult, a short adult, and adults with Parkinson’s disease and/or any other movement inhibiting conditions. Machine learning should be used to refine algorithms.

### 3.10. Managing Change

As reported by participants, digital transformation is process transformation. This must be acceptable to care staff at the residential home and staff must be trained in relation to new ways of working and the associated use of new technologies as part of care delivery. 

Overall, participants proposed that the minimum requirement for this technology would be to promote awareness about resident wellness, and to monitor and to report on caregiver action to manage resident wellness.

## 4. Emerging Concept

### 4.1. Overview of Sensing Solution

The primary objective is enable a positive resident and caregiver experience, as part of this, to support relationship-centered care. Accordingly, a suite of interrelated technologies (including tablets, desktop applications and a sensor kit) has been advanced for older people and other stakeholders (i.e., nurses, care assistants, admissions/administration personnel and family members), facilitating the gathering, integration and analysis of data across the three sensing dimensions. This includes (1) the resident and resident wellness, (2) the resident’s environment, and (3) care delivery.

Tablet and desktop solutions have been advanced for (a) residents, (b) care givers, (c) family members and (d) other actors (for example, maintenance personnel, concierge, entertainment coordinator), to enable all actors participate in care. For more information, please see Appendix E.

In relation to (a), as every resident is unique and has specific requirements and abilities, needs assessment and customization is the first step in relation to the implementation of the solution for individual residents. During admissions, the resident chooses what functions are available on the tablet, and how they appear on the user interface. Furthermore, the resident agrees to the sensor set-up—opting in and out of room sensors in line with their own preferences. The resident directly interacts with certain technology (for example, the resident tablet), while other technology is invisible (i.e., room sensors). Specifically, the resident tablet solution enables the resident to complete reports in relation to their wellness, contact family members, set-up social activity, view their social/activities calendar, access entertainment and view health information. In relation to (b) a suite of tools for different care-givers (doctor, nurse and care assistant) has been introduced. This link to stakeholder work activities as part of specific care processes including admissions, assessments, care planning, daily care and reporting events. In relation to (c) mobile solutions have been advanced to allow family members to track care and communicate with loved one and care staff.

### 4.2. Resident Profile and Resident Record

As stated earlier, a core requirement is to build a resident profile in relation to (1), (2) and (3), and to use the technology (tablet and sensor kit), to actively manage and update that profile, to optimize resident wellness. Resident profile information is captured in the ‘resident record’. Overall, the resident record captures relevant biopsychosocial information about the resident at different points in the care timeline. This information is derived both from the resident (i.e., inputs provided by the resident using the tablet), caregivers and other actors (who use additional tablet and desktop solutions) and sensors. 

The resident record anchors the whole system, connecting the workflows of (1) pre-admissions/admissions, (2) resident, (3) resident family members, (4) nursing staff, (5) care staff/nurse assistants, (6) concierge and (7) other medical staff. In relation to the currency of this information, a distinction is drawn between (a) static information (i.e., name, gender, age, interests, social profile), (b) dynamic information (i.e., health profile, health assessments, events and activities, goals and education, club memberships), and (c) real-time information (i.e., current location, health status, mood, pain, social activity, medication, food orders).

Table 3 below defines how the resident profile (and associated resident record) is used from the perspective of care processes and smart room technology/sensors.

### 4.3. Process Flow for Monitoring/Managing (1) Resident Wellness, (2) Resident Environment and (3) Care Delivery

The care-delivery process flow pertains to all three dimensions - namely, (1) resident wellness, (2) room and broader home environment, and (3) care delivery.

**Figure 3:**
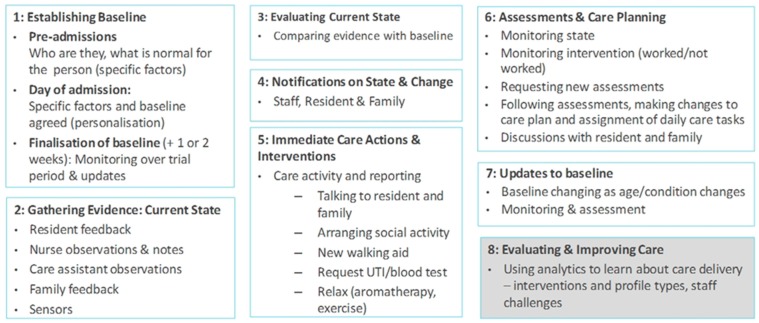
Process flow for monitoring/managing (1) resident wellness, (2) resident environment and (3) care delivery.

Figure 3 below provides an overview of the overall process flow for care delivery. As indicated in Figure 3, salient information pertaining to resident identity and health is captured at the admissions stage. Following relevant assessments, and a preliminary transition period (i.e., once the resident has become familiar with the new environment and established a daily routine), the health baseline is formalized. This baseline concerns all three pillars of resident wellness. Feedback about resident wellness is routinely captured as part of care-giver reports, resident reports and any reports from family members. This feedback is also integrated with sensor feedback (for example, information about sleep, activity and any biomedical parameters that are being captured). Caregivers and family members are notified, if there is a change from the baseline. This links to specific care processes to ascertain the reasons for such change, and what remedial action is required. Interventions are then made and reported (using tablets). The resident state is monitored to investigate whether the intervention was successful, and/or whether further interventions and/or follow up health assessments are required. This is undertaken in collaboration with the resident and their family. Depending on the outcome, there may be a requirement to refine the baseline, update care plans and assign new daily care tasks. 

In relation to the resident’s environment, the overall concept is to use the sensor kit to (a) enable resident control their room and/or (b) to optimize room setting to the resident’s preferences, routines and/or behaviors. Sensors are used in the broader living areas, but these simply record presence and movement, and enable free movement/access as is appropriate to the resident’s profile.

An example scenario is as follows:

- In relation to (1) resident wellness: A wellness report is completed by the resident using the tablet solution (specifically, the resident reports on sleep and mood problems);
- In relation to (2) resident’s environment: The resident’s bed sensor indicates certain changes to sleep patterns (i.e., out of bed more often and less sleep time);
- In relation to (3) care activity: The caregiver reports on resident rounding using the tablet solution—noting that the resident is fatigued, and that their mood has worsened;
- In relation to (2) resident environment: The room temperature in the room is lowered, and the speakers are set up to play calming music close to the resident’s sleep time;
- In relation to (3) care activity: The nurse prescribes daily physical and social activity to address sleep feedback from the resident, nurses and sensors, and mood feedback from self-reports and nurse observations. The nurse continuously monitors feedback from (1) and (2), to see if the resident’s sleep has improved and if there is an improvement in mood. This involves checking data in the nurse tablet pertaining to sensors and resident reports, along with an in-person conversation with the resident and/or their family. Depending on the outcome, there may need to be further assessments, and potentially a change in health baseline. This would trigger a new care plan, and revisions to daily care tasks and associated reporting of daily care.

The proposed technology has been designed to support different levels of intelligence, which can be (1) customized to the requirements of the care setting, and (2) incrementally implemented as part of a change process. Table 4 below presents the different levels of wellness management intelligence available. 

### 4.4. Wellness Indicator

**Figure 4:**
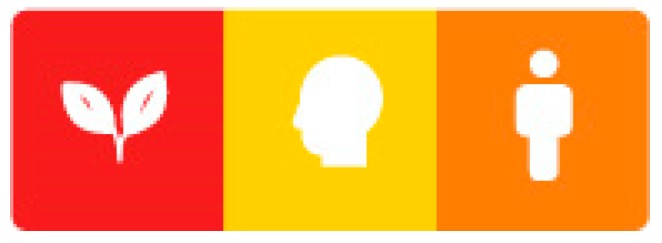
Wellness indicator.

As indicated in Figure 4 below, a wellness indicator has been advanced, which provides feedback to different stakeholders about the high-level state of the resident, in relation to the three wellness pillars. The objective of the indicator is to support:

1. Predictive risk management—flag wellness problems so they can be addressed early;
2. Staff briefing and handover in relation to resident wellness—daily summaries;
3. Resident/staff communication and care delivery;
4. Resident self-management of health (i.e., awareness of state).

The symbols are designed to represent each of the three pillars—namely, biological, psychological and social. The color coding on the indicator reflects a change in state, in line with the ‘traffic lights’ system used in aircraft cockpit displays. A difference in color reflects the level of change in state for any given pillar, and the associated severity. Ideally, residents will remain in the green, however, at different times, one or more pillars may be ‘under threat’ and displaying as either yellow, amber or red. 

The overall objective of the indicator is to communicate to residents and caregivers, what the current wellness state is and whether there is a change from normal (i.e., the resident’s baseline), so that remedial action can be taken. Table 5 below demonstrate the difference in appearance/color coding on the indicators, and the recommended wellness action. 

**Figure 5:**
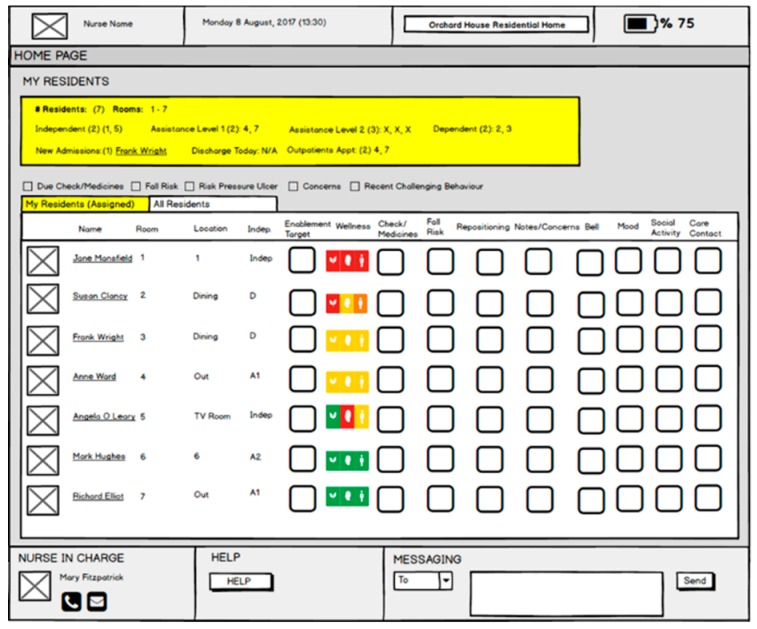
Prototype of dashboard of nurse tablet solution featuring wellness indicator.

The above wellness indicators feature on relevant tablet systems for residents, caregivers and family members. Figure 5 below provides an example in relation to the nurse tablet system.

## 5. Technology Overview

### 5.1. Overview of the Technical Architecture and Data Platform

**Figure 6:**
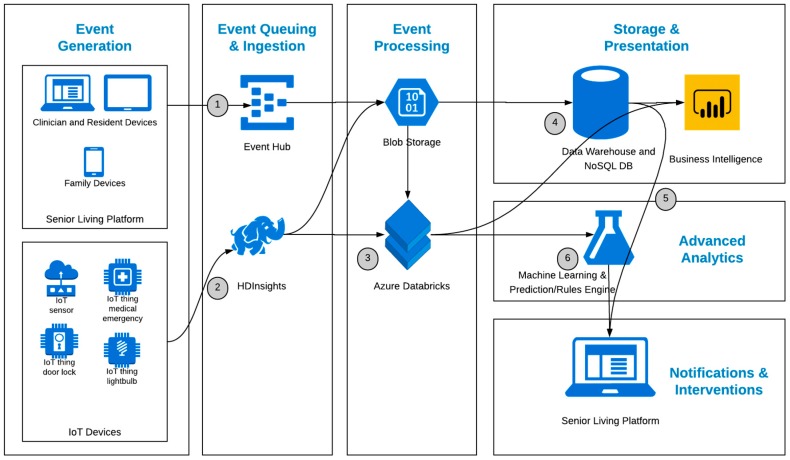
High-level technical architecture.

As the physical hardware of different sensor types is changeable and commodity based, the ethos of the product architecture is to abstract information from any specific piece of hardware or protocol. The platform is built with an IoT gateway which can receive data from any sensor kit and stream this data to the analytics platform in the cloud. Figure 6 below provides an overview of the product technical architecture. 

As indicated in Figure 6, data is generated by the interrelated technologies (tablets, desktop applications, and sensors), ingested and processed by the data platform, and then made available to the applications for further use.

(1) The resident and clinician devices access the senior living platform via apps on the tablets and desktop machines, which generate events that are processed by the senior living platform to perform operational functions, but also passed to the data platform event hub for reporting and analytics.
(2) The IoT sensor data is sent either aggregated or raw to the IoT hub and then HDInsights (Hadoop), which is another entry point into the data platform.
(3) The IoT and event data is processed in two key ways:
  - Run against rules-based logic to assist with operational decisions in the senior living applications
  - Processed and stored in a data warehouse for reporting and heavy computation analytics
(4) The data platform is hosted in Azure, so Azure tools are the default choice when technically feasible and not cost-prohibitive. Azure options for handling the above scenarios include:
  - Azure HDInsight for the IoT data processing [80];
  - Azure Databricks for combining the IoT data and application event data, cleaning it, and applying machine learning techniques [81];
  - Stream analytics for real-time analysis and pattern recognition of IoT and event data [82];
  - Logic Apps for rule-based triggers back into the senior living platform [83]
(5) The data from any of these sources can be returned as actionable information to relevant users, in an easy to understand form, allowing for the enhanced provision of care by giving the care user access to this information at the point of care.
(6) Machine learning algorithms deployed in a prediction engine can leverage the historical data in the multiple storage sources to train their models and make decisions against the real-time data flowing in.
(7) These decisions are what drive the “smart” capabilities of the system as outlined in the Sensor Kit and Sensor Profiles section.

### 5.2. Data Analytics

The initial phase of the data platform stores the data in Azure blob storage and a physical SQL data warehouse. This enables traditional reporting using tools such as Power BI for staff and management to perform recurring reporting such as daily, monthly, and quarterly reporting.

The blob storage is the source of data for the physical data warehouse, but it is combined with the IoT data stored in HDInsight via Azure Databricks for more complex analysis. This is stored in a NoSQL database and made available to the Senior Living applications.

Much of the data processed by the Data Platform (i.e., exclusive of raw unstructured data consumed by sensors) will be structured and frequently time-series, geo-spatial, or both. For instance, every event from the senior living application such as a resident assessment will include a timestamp when the assessment is completed, the location of the assessment, along with the structured data documented in the assessment and any unstructured text data.

The data platform can leverage a variety of machine learning techniques and tools based on the sensors available and the goals of the organization. For example, deep learning models and in particular, multi-task learning, can be used to assess fall risk on the basis of wearable sensor data [84]. However, not all organizations will use wearables such as GPS insoles. Accordingly, fall risk prediction may require the implementation of more traditional rules-based falls risk assessments (including the outcomes of functional tests of mobility and questionnaires undertaken in a clinical setting).

### 5.3. Implementation of Internet of Things (IoT) Environments

As IoT implementation depends on what sensors are available to the vendor/selected by the vendor, implementation is not locked to a given technology.

Several options are recommended for the transport layer (i.e., communication between sensors and IoT Hub, that is, layer 4 of the Open Systems Interconnection (OSI) stack model [85]. In order of preference, this includes:

- Thread (i.e., OpenThread) [86];
- Use of the standard TCP/IP stack;
- Radio frequency based (i.e., Zigbee) [87].

For the event bus, all recommended options are based on the concept of publish subscribe architecture. In this concept, sensors publish the data, and the IoT hub subscribes to them and stores them. In order of preference, the options include:

- Message Queuing Telemetry Transport (MQTT) which is the most common industry standard [88];
- Google IoT Cloud, specifically the Pub/Sub component [89];
- Proprietary solutions such as PubNub [90].

Often vendors lock the access to their IoT event bus and provide a gateway that stores the sensor data. In this instance, access is provided via a REST API. In such situations, the Oneview IoT hub can periodically poll the API to gather new data. New events are then stored in the Oneview database for machine learning and further processing. This second option lacks the real-time component of a publish service bus. A common solution for the lack of real-time is the definition of alerts and registering webhooks for these alerts, so that the vendor’s hardware will proactively contact the IoT hub when something happens that requires real-time intervention (for example, a door is opened, a smoke alarm is triggered, or a fall is detected).

From the perspective of data storage, almost any database can be used. Specific consideration is given to what makes most sense in terms of the data analytics platform.

Two proof of concept (PoC) implementations were undertaken. The first involved the creation of an event bus that was stored and registered. This was based on KNX hardware [91]. The second involved the application of custom sensors using Arduino, TCP/IP, PubNub and Firebase.

### 5.4. Technical Architecture and Data Security

**Figure 7:**
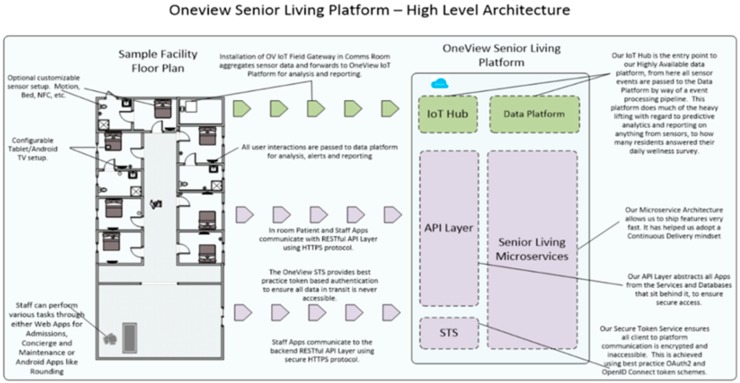
Technical architecture and security.

As indicated in Figure 7 below, all user interactions are passed to the data platform for analysis, alerts and reporting. Resident and Staff apps communicate with the RESTful API Layer using a HTTPS protocol. Secure token service (STS) provides best practice token-based authentication to ensure all data in transit is never accessible. The STS ensures all client to platform communication is encrypted and is accessible. This is achieved through QAuth2 and Open ID connect token schemes. The API layer abstracts all Apps from the services and databases that sit behind it, to ensure secure access.

### 5.5. Technical Architecture and Enterprise-Level Scalability

The architecture that underpins the Senior Living platform is designed to support enterprise level scalability. Several elements of the architecture enable this. This includes

1. Event sourcing;
2. Automatic horizontal scalability;
3. Couchbase Server Read Model, Couchbase Sync Gateway and Couchbase Lite;
4. Cloud availability;
5. Network disruption on site.

In relation to (1), event sourcing is used as the primary data store. This simplifies data storage and enables read models to be built that map directly to the query needed by a web or android client application. This ensures that database queries do not have to be written against third normal form or EAV-type data models, which often suffer under high load or volume. Instead a ‘denormalised’ view of the data is used. These views can also be rebuilt as new features are added. In this way, the data model can change as new features are added.

In relation to (2), we leverage Azures VM Scale Set capability along with the deployment tool Octopus to support auto-scaling to meet the demand of peak times such as shift changes and other peak events.

In relation to (3), the event sourced architecture pushes all data to a secondary NoSQL database called Couchbase Server. From here data can be grouped into a dedicated channel for each facility. Couchbase Sync Gateway is then used to sync all data related to residents to all devices in each facility’s dedicated channel. This ensures that data is only synched for all active users in a single facility to a single device. The primary benefit of this is that most requests for data by the care team are made against a local Couchbase Lite database on the Android device itself, rather than calling out to the cloud each time.

In relation to (4), the cloud-hosted solution requires no on-site changes to the server architecture and is fully scalable. Built on Microsoft Azure, data is protected by Microsoft’s comprehensive security measures. Several Azure features ensure a highly available platform. These include:

- Zone redundancy storage (ZRS) ensuring that all data stored on disk in our cloud is distributed across multiple zones in a region.
- For SQL database read models, Azure SQL databases inbuilt functionality is used.
- The Azure load balancer is geo redundant within zone.
- Each node behind the load balancer sits inside a scale-set that ensures nodes are distributed across multiple availability zones.

Access to the Oneview system is available via web applications and Android apps. Android apps are accessed via Android tablet devices (as per agreed hardware specifications). 

In relation to (5), as all state changes are pushed to all facility devices in real time, it is possible for all staff to continue to use the apps during times of network inactivity.

### 5.6. Sensor Kit and Sensor Profiles

The objective of the sensor kit is to track wellness indicators, to provide safeguarding for the resident in their living area, and to provide predictive analytics to care teams based on evidence pertaining to the resident’s activity and behavior. 

A passive sensor solution forms part of the resident’s environment. In terms of the resident’s room, this includes PIR activity-based sensors, bed sensors and sensors on doors and windows. All rooms feature access controls and intuitive signage (i.e., pictures of residents). Smart lighting/heating is used in resident rooms. Emergency call buttons are located by the resident’s bed and in the bathroom. Pending resident/family agreement, residents may wear GPS insoles (i.e., enabling safe and free movement where there is a risk of wandering). These can be tied into proximity locks at restricted areas (i.e., depending on the resident profile/permissions, the doors lock upon approach). Digital signage is used in communal/social areas. Sensors feature on all windows and doors around the facility. Real-time location systems (RTLS) are also used (i.e., tracking resident/staff locations). 

Machine learning is utilized to refine the idea of what counts as “normal behavior” for the resident. For example, an individual may routinely get out of bed to sit at the window to pray at times during the day, including night time. Machine learning would provide intelligence to ascertain that this is normal behavior for the resident, and a notification does not need to be provided to caregivers. However, for other residents, leaving the bed, and not moving and/or returning to the bed would generate a call to action.

Lastly, data analytics technology monitors and provides feedback about (1) resident wellness and activity (i.e., pain, sleep, falls, help requests, social activity, physical activity) and (2) staff activity (i.e., providing metrics in relation to care delivery /responses and feedback about the requirements for care assessments and revised care plans for specific residents).

The resident is assigned specific sensor profiles at various stages of the care spectrum, to ensure that the sensors are appropriate to their care needs. For example, if a resident has a history of wandering behavior and the sensors detect that they are getting out of their bed at night and leaving their room, then this would be flagged to the care team so that the behavior can be addressed before it escalates.

More general sensor profiles would address the issues of falls. For example, at night, bed sensors and PIRs can observe that the resident has got out of bed. This event would turn on low-level lighting to the bathroom to reduce the risk of a fall. The bathroom PIR would detect that the resident has entered the bathroom. If the bedroom PIR and bed sensor do not pick up that the resident has returned to their bed within a time parameter, then a notification can be provided to caregivers to check if the resident has fallen.

### 5.7. Customer Implementation and Sensors

As defined previously, Study 3 has involved two initial customer implementations in aged care settings. The first of these (i.e., customer 1) involves sensors and an IoT hub. The system incorporates a resident behavior profiling monitoring system to be integrated with the Oneview Assisted Living Platform. 

Resident behavior profile monitoring is a system of non-intrusive room sensors which utilizes the latest in Thermal sensor technology to monitor the individual behavior of each resident. The system continuously monitors the resident’s room for all activity. This includes real-time monitoring of the following factors:

- In bed
- Out of bed
- In bathroom
- Falls
- Multiple persons
- Intruder

Intelligent algorithms analyze and learn pattern recognition to identify the position of the resident. This enables live monitoring of physical activity, stumbling, falls, temperature changes, toilet visits and sleepin patterns. This information is used to identify hazardous hotspots (pattern of stumbling or falls), so that remedial actions can be taken. It is also used to identify trends in relation to room visits (including both care teams and family members). This can support analysis in relation to social and care activity. In the case of fall detection, short message service (SMS) alerts are provided to the care team, so that the resident obtains immediate help. 

**Figure 8:**
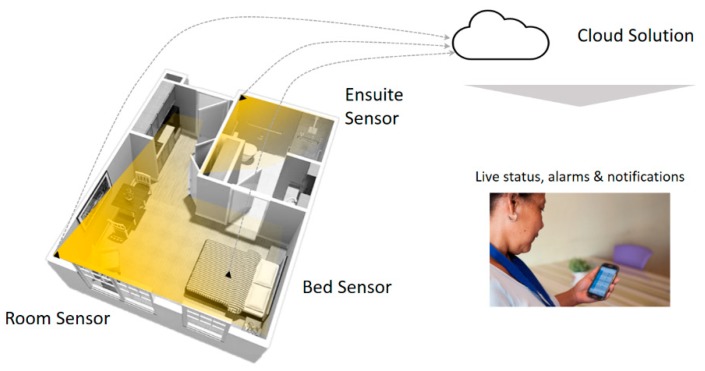
Overview of sensor system.

**Figure 9:**
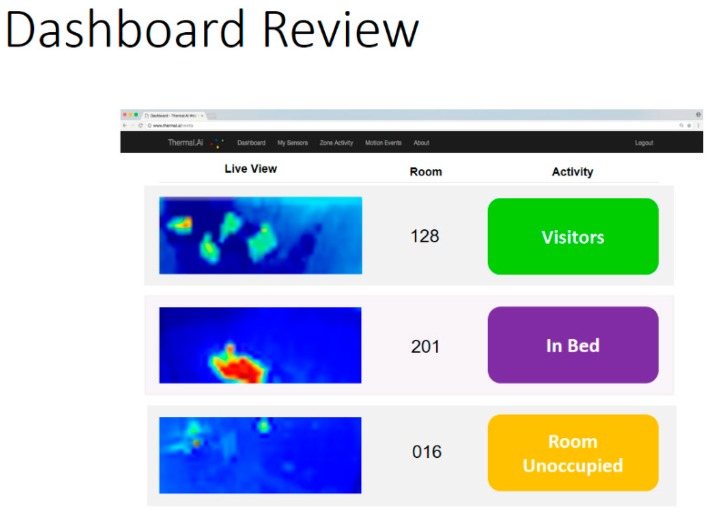
Dashboard review.

**Figure 10:**
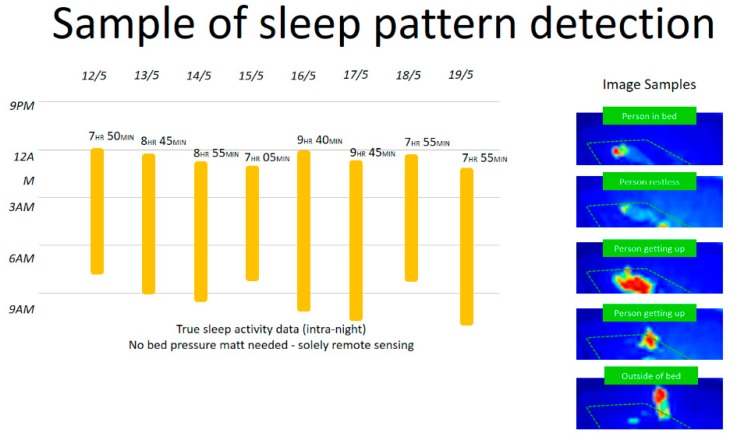
Sample of sleep detection.

**Figure 11:**
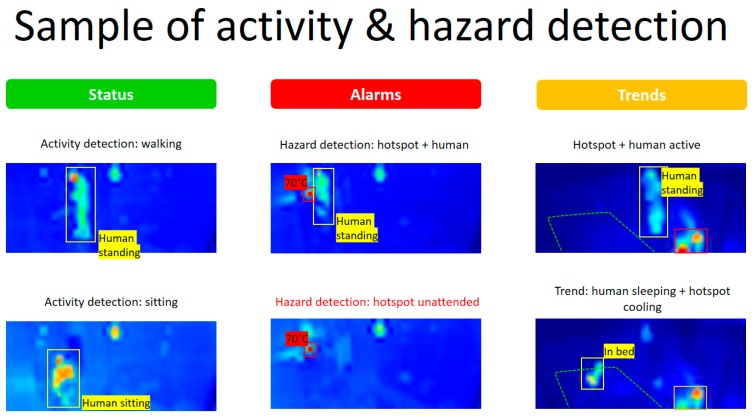
Sample of activity and hazard detection.

As indicated in Figure 8, the three sensors (i.e., the bed sensor, the room sensor and the ensuite sensor) are hardwired to a local power source (5V DC). Each sensor can either use the Wi-fi available or connect via the local area network (LAN). The IoT Gateway is located on each floor and is connected to the devices via the same network virtual LAN (VLAN). The IoT gateway then reports up to an Azure Instance for reporting and management. For more information on the three sensors, please see Appendix F. Figure 9, Figure 10 and Figure 11 below provide examples of the reporting output. This includes the dashboard overview, sleep patterns and activity and hazard detection.

In this instance, the customer has locked the access to their IoT event bus (i.e., they use publish/subscribe internally and provide a gateway that stores the sensor data). Access is provided via a REST API and routine polling is required.

## 6. Discussion

### 6.1. Structuring Device/Frame of Reference—Three Levels

The starting point for thinking about technology need is the concept of ‘living at home’—albeit in this case, the home is a site for care support and involves community dwelling. To this end, it is argued that the residents experience can be understood and monitored from three perspectives. This includes:

1. The state of the resident considering the three wellness pillars, with a key emphasis on social participation (including the resident’s relationship with caregivers, other residents and family members).
2. The state of the resident’s living environment (i.e., room and use of space in residential care facility), and how it is experienced by the resident.
3. The state of care delivery (i.e., whether the person has received care/assistance in accordance with their care plan and associated assessments).

**Figure 12:**
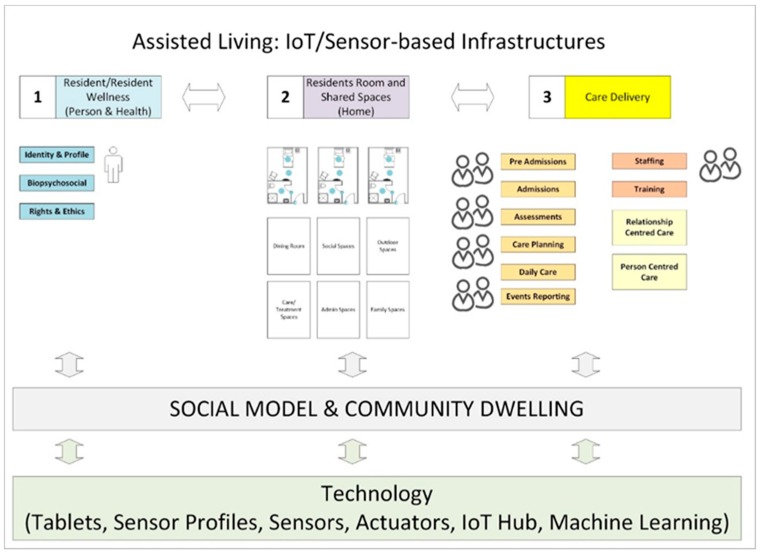
Internet of Things (IoT)/Sensor based infrastructures and 3 structuring principles.

As indicated in Figure 12 below, human/social communication underpins (1), (2) and (3); that is, it is implicit in both the (1) lived experience of residents and (2) the provision of care delivery. The proposed technology depends on this. Implicit in (1) and (3), is the resident’s relationship with caregivers, other residents and family members (i.e., communication and social relationship). This follows from concepts of relational autonomy, relationship centered care and person-centered care, as outlined previously. To this end, the proposed technology enables (a) residents, (b) family members and (c) all staff at the residential home to be involved in care.

### 6.2. Residents and Care Staff

Crucially, resident experience and staff professionalism are two sides of the same coin. If resources are scarce and if care-givers are undervalued and/or experiencing burn-out, then this will have an impact on care delivery. This in turn has consequences in relation to resident experience and resident engagement. To this end, the proposed solution needs to be designed to optimize the experience of both residents and caregivers. From a resident’s perspective, it needs to enable autonomy, social participation and quality of life. From a care-giver perspective, it needs to support care delivery addressing issues pertaining to workload management, teamwork, burn-out, compassion fatigue and establishing a rapport with residents.

### 6.3. Care Culture and Ethos

Primarily, the facility needs to be a home. It needs to be a place where residents thrive as opposed to decline. To do this, there must be opportunities for purposeful activity, personal growth (i.e., learning new things, participating in new activities), exercising personal choice, and strengthening of social relationships. Moreover, facilities need to address potential resident and family concerns comprehensively regarding the potential for loss of independence, encroachments on privacy, imposed routines and the potential for neglect. Moreover, older adults have real fears about being monitored (i.e., this includes both under monitoring and over monitoring). This is specifically salient in relation to the introduction of new technologies (i.e., permission for sensors, control over what information is shared with others and issues pertaining to a reduction in human contact).

### 6.4. Wellness Reporting and Care Delivery

Wellness reporting needs to consider multiple perspectives—including that of the person, family, care-givers and any information gathered from environmental and health sensors. As such, the proposed technology needs to be able to integrate the outputs of diverse reports, so that an overall assessment of resident wellness is provided. Further, the care process does not end at the assessment of wellness. Caregivers mush choose an appropriate action based on this assessment. Furthermore, all actions need to be monitored and evaluated, to determine whether the intervention is successful. This links into the overall care lifecycle—namely, establishing baseline, assessments, care planning and daily care.

### 6.5. Ageing, Identity and Technology

As we age there is a potential risk in relation to loss of identity. This is in part attributed to the inevitable experience of change (i.e., physical and cognitive) and loss which accompanies ageing. For example, aging can result in a loss of a role (for example, as a spouse), which may have been central to the older person’s identity [36]. This in turn can involve a confusion about one’s social role and often a sense of loss of continuity to one’s personality [37]. However, loss of identity is also linked to ageism (i.e., older persons perceived as having a lesser value and reduced capacity as they age). Questions of identity can be conceptualized on three levels. That is, in relation to a person’s (1) profile (i.e., whom am I now), (2) voice (i.e., role in decision making, user control and rights), and (3) social participation (i.e., being part of a community and having a role/purpose). Interestingly, there is a strong relationship between (1) a person’s profile, (2) their voice and (3) social participation. To participate socially, a person requires an identity (whom and I), which they can advocate for (have a voice/autonomy). 

If the proposed technology is to have a value for older people (and society more broadly), it must directly address issues around identity and identity preservation. Arguably, the proposed technology promotes identity on all three levels. The resident profile provides a picture of who the person is and what matters to them. This is supplanted with information about lived experience and state (framed from a biopsychosocial perspective). That is, what the person is doing now, how they feel and health status. The technology is underpinned by a rights base framework and concepts of user control (i.e., the person opts in/out of sensors and has control of their own information). Lastly, the technology promotes social participation—both within the residential home and with the broader community.

### 6.6. Societal Values

The advancement of assistive technology raises overarching questions in relation to the values of society and how we design technology to (1) promote positive values about ageing and (2) enhance ageing experience. Specifically, it raises fundamental questions in relation to the meaning of care and the role of people and technology in delivering care. This includes questions about what value we place on promoting autonomy and social participation for older people, protecting the personal sphere, and the importance of the human role in care (including family involvement). We should not proceed with this technology because it is available. Critically the human dimensions and care implications of this technology must be carefully considered.

Decision making regarding technology need and implementation must start from a principled basis. Specifically, technology might enable a situation where older people have quality care, have a voice/autonomy, where their privacy is respected, and where older people are proactive about enablement and social participation. In terms of care homes, the technology might enable care homes to be properly integrated in the community. Furthermore, the technology might enable all staff in the care home to be involved in care, as-well as families (if they so choose). 

Overall the technology needs to reflect a careful balance between optimizing the ability/strengths of the person while considering the needs (and workload) of caregivers. Certain information can be effectively gathered using sensors and resident feedback systems (i.e., surveys). This will allow more time for in person/communications. In this way, technology mediated care can be relationship centered. Importantly, this technology will not replace/supplant the need for ‘person to person’ contact.

### 6.7. Relationship Centered Care, Technology and Transforming Societal Values

It is argued that relationship centered care provides the framework for thinking about care, societal values and the technology role. Future IoT and sensor-based infrastructures needs to consider both (1) the resident and (2) enabling positive relationships and communications between residents and other stakeholders. The achievement of benefits in relation to resident experience is dependent on situating technology development in the context of enabling and fostering these relations. In this regard, the introduction of AAL technology serves to promote conversations about ageing experience, care for older people and what is acceptable. Specifically, new AAL technology can pave the way for improving the lived experience of older people. In this sense, the proposed technology might both enable and transform societal values concerning ageing and care for older people.

### 6.8. Research Status and Next Steps

Currently, this technology is being implemented at two customer sites. In relation to the first site, the proposed technology has just been installed and implementation feedback is pending. In relation to the second site, the technology has not yet been installed. In both cases, follow up human factors research will be undertaken to assess the lessons learned from the initial implementation. It is anticipated that this will provide additional insight as to what is acceptable to end users, how the technology is used in real operations, and usability issues. Moreover, it will provide information regarding challenges at an organizational level (including process change, resourcing and capacity and cultural issues) and technical challenges. This feedback is necessary to validate the proposed solution/product concept.

### 6.9. Study Limits and Areas for Further Thinking

As noted previously, this is an action research study. In relation to Study 1a, the initial requirements elicitation and co-design activities have focused primarily on the resident solution. Solutions for other stakeholders (i.e., nurses, care assistants and family members) have been addressed in relation to providing resident benefits. These solutions require additional definition.

Certain limitations in relation to the participant panel should also be noted. In relation to study 1a, the participant panel did not include older persons domiciled in residential homes. Nurses and family members provided indirect feedback in relation to the perspective of such residents. Furthermore, the participant panel in study 1b, included patients domicile at a post-acute care service.

Additional research is required with older adults with diverse age related physical, sensory and cognitive challenges. It is anticipated that this will be undertaken as part of the customer implementation evaluation.

Currently, considerable research is underway in relation to the application of voice interaction, for the resident tablet solution. It is anticipated that this simplifies user interaction for older adults, including older adults with vision issues and/or issues with manual dexterity (for example, hand stiffness and/or hand tremors). 

At a technical level, research is also addressing issues pertaining to decentralizing IoT networks and moving functionality to the edge. Potentially, this will involve the application of fog-computing models where IoT hubs are responsible for time-critical operations, while cloud servers take responsibility for data gathering and analysis.

Staff training is a key focus of the implementation plan. As noted previously, caregivers expressed a strong concern over the implications of this new technology for person-centered care. This is particularly salient in relation to staff usage of tablets while in the resident’s room. It is important that staff maintain eye contact and that the device does not negatively impact on resident/caregiver contact. It is anticipated that best practices will be established as part of the implementation program. That is, staff will provide feedback as to how this is working in practice, and work together in relation to establishing usage protocols. Furthermore, it is anticipated that this may also yield user interface design recommendations for specific care reporting forms. For example, certain changes may be required in relation to the layout and workflow for daily care and medications reporting forms, to ensure that staff can easily interact with residents and maintain eye contact, while at the same time interacting with the tablet to check resident information and/or report on care. 

## 7. Conclusions

This research provides a roadmap for the implementation of future AAL technology including sensor-based infrastructures in assisted living contexts. For assisted living facilities to be a home, we need to consider both the resident and the caregiver experience, and the allied technology requirements for each. Residential care facilities should provide a compassionate, social and ethical resident experience. Furthermore, they should provide a positive experience for care-givers. The digital transformation of certain care/clinical workflows offers opportunities to enhance care quality, resident safety and resident/care-giver interaction. Overall, the requirements for new AAL technology can be conceptualized on three related levels. This includes the state of person, the state of the home/environment, and the state of care delivery. The question of technology goes beyond tablets and sensors. It pertains to people, social participation, community dwelling, and care delivery experiences. Future AAL technology should be premised on biopsychosocial models of wellness, concepts of home and support relationships between older adults and members of the personal and professional community. The proposed technology affords the possibility for improved social relationships, enhanced wellbeing, better quality of care, and independence. However, such technologies require careful consideration in relation to adapting to age/condition and managing issues pertaining to resident consent, privacy and human contact.

## Figures and Tables

**Table 1:** Studies and research themes.

#	Theme	Subtheme	1a	1b	2	3
3.2	Resident Experience	Concept of home	*	*		*
		Residential home	*	*		*
		Comfort	*	*		*
		Resident states	*	*		*
		Identity social participation	*	*	*	*
3.3	Care Delivery	Scope and processes	*	*		*
		Wellness communications	*	*		*
		Wellness and stability	*	*		*
3.4	Residential Care and Sensing Framework		*	*	*	*
3.5	Technology Goals		*	*	*	*
3.6	Ethics and User Acceptability		*	*		*
3.7	Application of Existing Sensor State of the Art to Residential Care	General			*	*
		Sensors and monitoring person/resident			*	*
		Sensors and monitoring environment			*	*
		Sensors and monitoring care delivery			*	*
3.8	Technical Architecture				*	*
3.9	Design		*	*	*	*
3.10	Managing Change		*	*		*

**Table 2:** High-level technology goals.

#	High-Level Technology Goals
1	Enable holistic care delivery—underpinned by concepts of holistic care—attention to wellness, relationship centered care and professionalism.
2	Overall, use technology (sensor and tablet kit) to build a resident profile. This includes a picture of the (1) resident and their wellness, (2) how they are living in the environment, and (3) their care, and (4) the relationships between each of these.
3	Following from (2), to use the technology (tablet and sensor kit), to actively manage and update the resident’s profile, to optimize resident wellness.
4	Link up information flows arising from the diverse care processes—admission, assessments, care planning, daily care, reporting, adverse events reporting.
5	Predictive risk management in relation to resident wellness and stability—anticipate state changes and flag need for interventions if required.
6	Continuously monitor status of care delivery—if missed rounding or medication—and notify care-givers and management as to status.
7	Flag need for interventions at environmental level (adjust room lighting, temp etc.), and automate action to ensure room settings appropriate to resident preference and/or wellness.
8	Support staff communication (staff briefing and handover).
9	Support resident/staff communication and care delivery.
10	Enable everybody involved in care/report on resident wellness (resident, family, nurse, Dr, care assistant, admin).
11	Gather data about (1) individual residents, (2) all residents—to improve care planning/quality of care.

**Table 3:** Sensing framework and resident profile.

#	Sensing Framework	Care Processes			Sensors and Smart Room
		Admissions	Assessments and Care Planning	Daily Care	
1	Resident & resident wellness	Advance initial personal profile Advance preliminary health profile	Following assessments, define a health profile and baseline in relation to key wellness parameters (biological, psychological and social)	Track and report wellness against baseline If significant changes, identify need for reassessments and new care plan (i.e., relationship to 3)	Advance sensor profile for resident linking to (1) Track health/wellness including social and physical activity
2	Environment	Define resident preferences for room	N/A	N/A	Over time, use sensors to build profile of resident/continuously learn about person and room preferences and relationship between wellness and room settings Make changes to room settings, based on learnings
3	Care Delivery	Establish preliminary care need linked to health profile (1)	Following assessments, assign a care profile and specific care tasks	Report on care delivery Track receiving care in line with care plan etc	Track human contact/presence of caregiver in room

**Table 4:** Wellness management and technology intelligence levels.

#	Wellness Management
1	Residents and care assistants reports on wellness and wellness information is available to all (integrated in existing reports, surveys, specific wellness reports).
2	Includes 1 Also, staff report on actions taken to manage wellness.
3	Includes 1 and 2 Also, system automates a basic summary wellness evaluation/status (for 3 pillars)—based on analysis of parameters provided by ALL (resident, nurse, care assistant, family).
4	Includes 1, 2 and 3 Also, system recommends care actions (spanning 3 pillars), which can be accepted, or alternatives proposed—all actions documented by staff using system.
5	Includes 1, 2, 3 and 4 Also, system requests reports from relevant actors on status of care actions—if successful/improvement in wellbeing.
6	Includes 1, 2, 3 and 4 Also, system provides intelligence as to care outcomes, requirement for reassessment, other interventions.
7	Wider reporting and analytics over time for care facility, per issue, per intervention, per patient.

**Table 5:** Wellness Indicator, Change in State & Care Actions.

#	Change in State	Care Action	Indicator
1	No Change	None	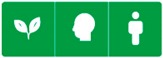
2	Minor Change	Monitor	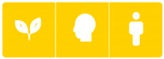
3	Significant Change	Action Required	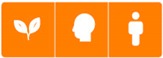
4	Major Change	Action Required—(Immediate/Urgency)	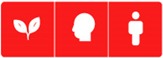

## Appendix A. Overview of Study 1a Research Phases & Activities

**Table A1:** Overview of study 1a research phases and activities.

Phase	Research Phase/Activity	Stakeholders	No of Participants	Personae	Prototype
1	Literature analysis	N/A	N/A	N/A	N/A
2	Preliminary Definition of Approach, concept, high level requirements and personae	Internal stakeholders	N = 3	Personae 1	N/A
3	Needs/Requirements Elicitation (interviews and observations with stakeholders)	Internal and External stakeholders	Internal: N = 9 External: N = 38	N/A	N/A
4	Needs/Requirements Elicitation (observations)	External Stakeholders	N/A	N/A	N/A
5	Needs/Requirements Analysis: Elaboration of concept and philosophy and specification of detailed requirements and personae	Internal stakeholders	N = 2	Personae 2	N/A
6	Production of Initial Prototype	Internal stakeholders	N = 2	N/A	Prototype V 1
7	Co-design and Evaluation, Phase 1	External stakeholders	N = 6	N/A	Prototype V 1 and V 2
8	Co-design and Evaluation, Phase 2	External stakeholders	N = 5	N/A	Prototype V 2 and V 3
9	Needs/Requirements Elicitation (Residential Home Study)	External stakeholders	N = 5	Personae 3	Prototype V 3 and V4
10	Co-design and Evaluation, Phase 3	External stakeholders	N = 5	N/A	Prototype V 4 and V5
11	Co-design and Evaluation, Phase 4	External stakeholders	N = 5	N/A	Prototype V 5 and V6
12	Co-design and Evaluation, Phase 5	External stakeholders	N = 5	N/A	Prototype V 6 and V7
13	Final Specification and Design	N/A	N/A	Final Personae	Prototype V 8
Internal stakeholders = members of project team External stakeholders = older persons, nurses, family members, dementia experts, residential home managers/owners and gerontologists					

## Appendix B. Overview of Study 1a Methods

**Table A2:** Overview of study 1a methods.

Method	Description
Literature Review	The literature view focused on providing a qualitative summary of existing evidence pertaining to successful ageing, wellbeing, care approaches, stakeholder need, technology requirements and ethical issues. Relevant theoretical literature pertaining to successful ageing, wellbeing and care approaches was reviewed. Policy documentation and research studies pertaining to the advancement of assisted living technologies were also examined. Furthermore, an analysis of the existing competitor offering was undertaken. Lastly, the researcher reviewed requirements documentation provided by three prospective customers.
Needs/Requirements Elicitation (interviews)	Semi-structured interviews were conducted in person either at the participants home or their workplace. Four separate interview guides were developed to support interviews with (1) older adults, (2) family members, (3) nurses/care staff and, (4) ageing experts and volunteers. Specific interview questions linked to key research questions and relevant themes emanating from the literature review. In each case, the participant was posed questions pertaining to their own experience and needs, and that of other stakeholders. Overall, 47 interviews were conducted with external (N = 38) and internal (N = 9) stakeholders. In terms of interview duration, 25 long interviews (approx. average duration 2 h) and 22 short interviews (approx. average duration 0.5 h) were undertaken. In all cases, the researcher took written notes. There was no audio or video recording. In terms of participant breakdown, external stakeholders included 11 older adults (mean age mean age 79.36 years), 7 family members, 5 experts in ageing/dementia, 1 ICT expert, 4 nurses, 5 representatives spanning two ‘care for the elderly’ day services, 3 representatives from a post-acute care service, and 2 representatives from a residential home.
Needs/Requirements Elicitation (observations)	Preliminary observations were undertaken taken at two day hospitals and one residential home. A short walk around was undertaken at one day hospital (approximate time: 0.5 h). The researcher was accompanied by the assistant director of nursing. A detailed walk-around was undertaken at a second day hospital (1 day). Here the researcher was accompanied by the nursing manager, along with other staff (dietician, case manager and pharmacist). The researcher observed several settings—case rooms, occupational therapy room, service user interviews and assessments and service user social activities/interaction in the common room. Furthermore, the researcher received a walk-through of relevant technologies used by different staff, to document case work. A third observation was undertaken at a residential home (approx. time 2 h). Here, the researcher visited indoor and outdoor social spaces, dining rooms, activity rooms and resident rooms accompanied by the residential home owner. In all cases, the researcher collected artefacts from the settings including paper forms and information leaflets. In relation to the second day hospital, the researcher took screenshots of the technologies used by the different roles, and schedule/workload information presented on nurse whiteboards.
Needs/Requirements Analysis: Elaboration of concept and philosophy and specification of detailed requirements and personae	Following the interviews/observations, the researcher’s notes were transcribed. Data analysis focused on understanding/defining the lived experience of older adults, care approaches and specific stakeholder technology requirements. A thematic analysis of field research findings was undertaken. The thematic analysis was initially driven by the research question and associated theory (i.e., not inductive). In support of this, a preliminary coding/data frame with high level nodes was defined. An initial review of sample manuscripts across different stakeholders was undertaken. Following this, the coding frame was refined, and sub-nodes were identified (i.e., link to emergent themes).
Production of Initial Prototype	Stakeholder personae and scenarios were used to (1) support problem solving around stakeholder requirements and (2) the user interface design of prototypes. Early stage prototypes were developed using the wire framing tool Balsamiq. The primary focus was on the resident solution. Three different prototypes linking to three high level resident contexts were defined: (a) resident is considering moving to residential home, (b) resident is in the admissions process and (c) resident is domicile in residential home. The specification of (c) involved the parallel definition of complementary prototypes for other stakeholders. This includes prototypes for nurses, care assistants and family members. The initial prototype was reviewed by members of the project team (internal stakeholders, N = 2), in advance of the co-design activities.
Co-design and Evaluation Phase 1	The first phase of co-design/evaluation occurred after the analysis of field research and the specification of the initial prototypes (prototypes 1). The evaluation focused on eliciting stakeholder feedback regarding the high-level product concept for the resident application, and a subset of the other stakeholder solutions (for example, nursing staff, families, pre-admissions and admissions). External stakeholders included older adults (N = 4) and nurses (N = 2). In advance of viewing the prototypes, participants reviewed a short Microsoft power-point presentation which provided a background to the research and preliminary findings, a summary of the different applications and functions, and an example persona. The review/co-design of prototypes then commenced. The initial prototypes were demonstrated to stakeholders using a laptop computer. Participants were invited to review prototypes based on (1) their own experience and need, and (2) on the imagined situation of Frank (persona). Feedback pertaining to user need, user expectations, user acceptability and issues related to ethics and privacy was elicited.
Co-design and Evaluation Phase 2	The second phase of co-design involved the same procedure as phase 1. However, the second phase of co-design focused on the resident and nurse applications only. The prototypes demonstrate basic level functionality on the tablet (i.e., touch interaction). The focus here was on (1) eliciting more specific usability feedback (i.e., task workflows, interaction style, nomenclature and iconography) and (2) following up on certain key human factors issues (for example, reduction in human contact). Participants included older adults (N = 3) and nurses (N = 2).
Co-design and Evaluation Phase 3	The third phase of co-design involved the same procedure as phase 1. However, this phase focused on the nurse applications only. Five nurses provided feedback about specific nursing workflows—pertaining to reviewing the overall resident status, assessing care needs for specific residents, reporting on daily care and monitoring wellness interventions.
Co-design and Evaluation Phase 4	The fourth phase of co-design involved the same procedure as phase 1. This phase focused on refining the concepts, processes/workflows and specific user interface (UI) design for both the resident and nursing applications.
Co-design and Evaluation Phase 5	The fifth phase of co-design involved the same procedure as previous phases. However, this phase focused on the nurse applications only. The primary focus was on (a) evaluating those functions which pertain to resident wellness (for example, reporting on resident status and activity, ensuring meaningful interactions with the resident, evaluating resident changes and making a case for new assessments/care plan updates), and (b) exploring how best to manage issues around reduction in human contact/optimizing human contact in care delivery. Five nurses provided feedback pertaining to specific nursing workflows.

## Appendix C. Three Pillars and Factors to be Monitored

**Table A3:** Overview of study 1a methods.

Pillar	Factor	Time-Period	Changes to
Biological	Mobility	24 h	Level of Mobility
	Activities of Daily Living (ADL) Support	Week	ADL Support/Dressing ADL Support/Feeding ADL Support/Toileting ADL Support/Hygiene
	Night Sleep	24 h	Not sleeping as normally do • Sleep (Bed Exists) • Sleep time
	Fatigue and Day Sleep	24 h	Sleep routine during the day (being in bed during day or level of sleep during day)
	Eating and Drinking	24 h	What eating How much eating How much drinking Refusing food/drink Issues with swallow
	Toileting	24 h	Changes to elimination/typical patterns (constipated etc.)
	Pain	4 h	Level of pain Comfort being dressed/showered
	Pressure Sores	24 h	Change in pressure sore status? New pressure sore?
	Body Temperature	2 h	
	Basic level Physical activity	Several days	Leaving bed/sitting out in chair Leaving room
	Physical Exercise	Several days	Taking walks outside Engagement in exercise activities
	Wandering	Several days	
	Falls	Week	No of falls
	Nurse Bell Requests	24 h	
Psychological	Mood	Several days	Difference in mood
	Cognitive	Several days	Sad, Anxious, Depressed
	General Behavior	Several days	Difference in
	Dementia Specific Behavior	Several days	Awareness
	Nurse Bell Requests	24 h	Understanding instructions
	Engagement: Hobbies and Interests	Several days	Memory (person, place, time)
	Engagement in Education	Several days	Difference in
	Engagement in Self-Management Activities	Several days	Level of stress, confusion, agitation
Social	Personality/Level of Social Engagement	Several days	Normally outgoing and talk to people—but change?
	Time in Room	Several days	Change in typical social patterns—not leaving room, spending more time alone than normal
	Club Events and Hobbies	Several days	Not responding to RSVPs Not attending/following normal patterns of attendance for club events
	Family Visits	Several days	Not having visits, refusing visits, cancelling visits
	Travel Outside	Several days	Not leaving facility—irrespective of whether out hours permissible
	Engagement: Communication with Staff	Several days	Changes in communication level Changes in communication style

## Appendix D. Examples of Wellness Communications

**Table A4:** Examples of Wellness Communication.

**Resident Behavior/Activity**	**Resident to Care Assistant**	**Nurse to Resident**	**Resident to Daughter**
Resident Fidgeting Resident spitting out food Resident Wandering Resident ringing call bell 7 times in 3 h	I’m not feeling well? When is Jane (daughter) coming to visit? What can I do today? Can I leave? I want to leave!	Hi, how are you feeling today? How is the pain? How did you sleep? Are you not hungry? Stroking resident’s arm and eliciting response	Is it you Jane (daughter)? The pain in my side is getting worse I can’t eat this dinner Where am I? Can I leave? I want to leave!
**Resident’s Daughter to Nurse**	**Between Staff Nurses and Care Assistants**	**Residents Nurse to Daughter**	**Room/Environment**
Mum seems in more pain It’s hard to tell whether mum is doing any better—what do you think? Mum seems off/not herself/not doing activities—could there be something wrong? Is mum falling more these days? Mums seems to be angry and doesn’t want to talk—what should I do?	How is Zena—any improvements? I’m having problems with room 10 Can you ask the Dr to have a look at Zena when he arrives? I need two people for room 10! Have we checked Zena for a Urinary Tract Infection (UTI)? I think we need to look at Zena’s care plan again.	Zena fell yesterday—her mobility is getting worse We think Zena might have a UTI and are doing some tests? We need to change Zena’s care plan Zena’s needs more care/help	Room/doors open

## Appendix E. Overview of Solutions for Different Stakeholders

**Table A5:** Overview of Solutions for Different Stakeholders.

#	Process	Actor	Application and Device	Description
1	Preadmissions	Sales Representative	Pre-admission app (website)	Resident and/or loved one provides background information about the resident—social and personal profile, health status, prior assessments and ability.
2	Admissions	Admissions Co-coordinator	Admissions App (Desktop Computer and Tablet)	The admissions application is used to complete the resident profile picture, at the time of admissions. Prior information provided at the preadmissions stage is prepopulated in the system. The admissions user interface is conceptualized in terms of a series of steps to promote familiarization for both residents and care staff, and reassurance for residents and family members.
3	Assessments and Care Planning	Nurse	Assessments App (Desktop Computer)	This records resident assessments information—comprising general wellness, nutrition, activities of daily living and functional ability, cognitive, behavioural etc
4	Daily Care	Resident	Resident App (Tablet)	The resident solution is customized in relation to resident need and ability. The resident can select from a series of functions—based on need/interest. Residents with mid to late stage Dementia—are not expected to interact directly with tablet system (require assistance).
		Nurse	Nurse Rounding App (Tablet)	The nursing solution promotes meaningful interaction with the resident, based on a real time picture of the resident’s state, and background information about who the resident is and what matters to them. This application is also used to record rounding information—structured from biopsychosocial perspective.
		Care Assistant	Caregiver App (Tablet)	This care assistant application enables reporting on ADL and caregiving tasks—it also provides access to information about the resident—personal history, what matters.
		Nurse Manager	Care Console (Desktop Computer)	Supports queries/data analytics in relation to resident wellness, assessments, activity and so forth. Also provides real-time information about the resident.
		Family	Family App (phone, web)	This allows the family member to view relevant real time and historical information about their loved one. Used also to upload content/information etc.
5	Maintenance	Maintenance	Maintenance Managements (Desktop and Tablet)	This application is used to process and manage maintenance requests from residents and staff.
6	Resident Activities and Entertainment	Entertainment Coordinator	Event Managements (Desktop)	Manage resident social activities and events.
7	Concierge	Concierge Manager	Concierge (Desktop)	Manage resident requests (i.e., travel, room maintenance etc).

## Appendix F. Study 3, Customer 1—Sensor Technology Implementation

**Table A6:** Overview of Room Sensors.

Room Sensor	Bed Sensor	Ensuite Sensor

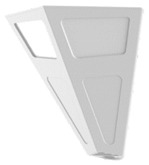
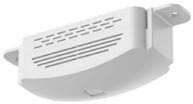
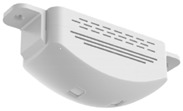
		
Complete room view for activity/wellbeing tracking and fall detection Mounted onto Corner of Room 5V DC 2.0A Max input via Micro USB Data transmission via Wi-fi or LAN	In-bed detection and advanced sleeping pattern analytics Mounted onto Ceiling 5V DC 2.0A Max input via Micro USB Data transmission via Wi-fi or LAN	Complete room view for activity tracking and fall detection Mounted onto ceiling 5V DC 2.0A Max input via Micro USB Data transmission via Wi-fi or LAN
